# Ecological Trap in the Nest: Human Hair Causes the Death of Breeding Barn Swallows (*Hirundo rustica*)

**DOI:** 10.1002/ece3.73592

**Published:** 2026-04-30

**Authors:** Cong Peng, Kui Yan, Sidan Lin, Wei Liang

**Affiliations:** ^1^ Ministry of Education Key Laboratory for Ecology of Tropical Islands, Key Laboratory of Tropical Animal and Plant Ecology of Hainan Province College of Life Sciences, Hainan Normal University Haikou China

**Keywords:** anthropogenic materials, barn swallow, human hair, nest material, plastic

## Abstract

As urbanization intensifies, an increasing number of bird species incorporate anthropogenic materials into their nests. This behavior may confer certain benefits but can also constitute an ecological trap, causing mortality through entanglement. In this study, we aimed to assess the prevalence of anthropogenic materials in barn swallows ( *Hirundo rustica* ) nests and their association with entanglement injury or mortality. We examined the nest material composition of 193 active barn swallow nests from two geographically distinct populations in China (Hainan in the south and Heilongjiang in the north), finding that 38.3% of nests contained anthropogenic materials, principally human hair (22.3%) and plastic (21.8%), with both occurring at significantly higher rates in the south Hainan population. We report, for the first time, seven cases (3.6%) of entanglement mortality in breeding adults and nestlings caused by human hair and plastic (fishing line) incorporated into nests. Although the number of individuals killed by entanglement in human hair and plastic was not large, our findings indicate that the ubiquitous presence of human hair and plastic in the environment may pose a direct threat to bird survival. A comprehensive, multidimensional understanding of the interactions between birds and anthropogenic materials will contribute to a broader assessment of the threats that environmental pollution poses to avian populations.

## Graphical abstract

The prevalence of anthropogenic materials in barn swallows ( *Hirundo rustica* ) nests and their association with entanglement injury or mortality was studied. We report for the first time that hair incorporated into nests can cause entanglement mortality in barn swallows. The ubiquitous presence of hair and plastic in the environment may pose a direct threat to bird survival.

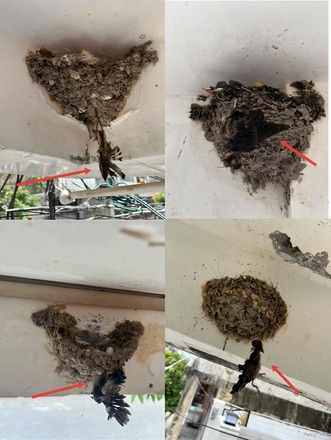

## 1 Introduction

It is estimated that the world currently generates 3 million tons of waste per day (Hoornweg et al. 2013). Anthropogenic litter is among the principal threats to natural ecosystems (MacLeod et al. 2021; Galgani et al. 2013). For example, airborne microplastics and nano‐plastics can function as cloud condensation nuclei and ice‐nucleating particles, influencing cloud formation and lifecycle and thereby altering precipitation patterns and global climate (Aeschlimann et al. 2022). The accumulating load of suspended plastic particles in aquatic environments increases the turbidity of cyanobacterial and phytoplankton habitats, reducing population sizes and altering community composition, which in turn reduces carbon sequestration from the atmosphere and contributes to global warming (Shen et al. 2020). In the soil, litter modifies key soil properties, leading to reduced water‐holding capacity, nutrient loss, and altered microbial activity and diversity (Bandopadhyay et al. 2018). Thus, anthropogenic waste permeates all major compartments of ecosystems, and a comprehensive understanding of its associated threats is essential for devising effective management responses.

Anthropogenic litter is also recognized as a threat to marine megafauna (Provencher et al. 2017; Heinze et al. 2025) and certain terrestrial animals (Plaza and Lambertucci 2017). The most commonly documented forms of interaction between wildlife and litter are entanglement and ingestion. A recent global synthesis recorded 994 marine megafaunal species, including 226 seabird species, 86 marine mammal species, all sea turtle species, and 430 fish species that have been reported to become entangled in or to ingest plastic (Kühn and van Franeker 2020; O'hanlon et al. 2021). A prominent example is abandoned fishing gear being the leading cause of mortality, after deliberate killing, in the endangered Mediterranean monk seal ( *Monachus monachus* ), of which only 600–700 individuals remain (Karamanlidis et al. 2008). Large‐scale, sustained removal of marine debris has been shown to reduce entanglement risk (Baker et al. 2024).

While anthropogenic litter threatens birds, many species have been observed incorporating foil, plastic strings, and pieces of clothing into their nests (Jagiello et al. 2019, 2023; Sheard et al. 2024; Rangel et al. 2025). This behavior may confer a range of potential benefits, including substitution for naturally scarce nest materials (Wang et al. 2009; Lee et al. 2015), structural reinforcement of the nest (Antczak et al. 2010), nest decoration (Borgia 1985), signaling of individual quality and territorial status (Sergio et al. 2011), reduction of nest predation (Husby and Slagsvold 2025), deterrence of ectoparasites (Suárez‐Rodríguez et al. 2013), and improved hatching success (Chen et al. 2024).

However, when the costs of incorporating anthropogenic litter as nest material outweigh the benefits, this behavior becomes an ecological trap (Dwernychuk and Boag 1972; Gates and Gysel 1978; Heinze et al. 2025). For instance, a review of the literature on anthropogenic materials in bird nests by Jagiello et al. (2019) revealed that entanglement had been recorded in 36% of species (24 species). Restani (2023) reported that 44.2% of osprey ( *Pandion haliaetus* ) nests contained plastic twine, resulting in entanglement of 3.4% of nestlings. Heinze et al. (2025) examined 568 white stork ( *Ciconia ciconia* ) nests and found that 91% contained anthropogenic materials, with nestlings entangled in 12% of nests. Despite these findings, existing research on anthropogenic nest materials is geographically biased, with the vast majority of studies concentrated in Europe and North America (both 32%). Only approximately 4% of such studies have been conducted in Asia (Jagiello et al. 2019). The broader literature on bird–litter interactions is similarly skewed: of 258 species documented to interact with anthropogenic litter, 206 species (79.8%) are seabirds, reflecting a strong focus on marine systems (Battisti et al. 2019; Heinze et al. 2025). Terrestrial species, particularly those in Asia, remain comparatively understudied (Battisti et al. 2019; Heinze et al. 2025).

To contribute toward filling these gaps, we examined the nest material composition of two geographically distinct barn swallow ( *Hirundo rustica* ) populations in China, one from the northernmost part of the species' Chinese range (Heilongjiang Province) and one from the southernmost part (Hainan Province). These two locations were selected to represent contrasting climatic and environmental contexts, as well as differing levels of anthropogenic pressure. Heilongjiang is a high‐latitude temperate region with low human population density, whereas Hainan is a low‐latitude tropical island characterized by high human population density. We hypothesized that the prevalence of anthropogenic nest materials (particularly human hair and plastic) would be higher in Hainan than in Heilongjiang, reflecting greater local availability of such debris in more densely populated urban environments. The main objectives of this study were to: (1) assess the prevalence of anthropogenic materials in barn swallow nests; (2) document cases of injury or mortality in adults and nestlings attributable to anthropogenic nest materials; and (3) compare nest material composition between two geographic populations.

## 2 Methods

### 2.1 Study Area and Species

One study site was located in Danzhou City, Hainan Province (19°11′–19°52′ N, 108°56′–109°46′ E), in the northwestern region of Hainan Island in south China. The site has a tropical monsoon climate, with a mean annual temperature of 23.1°C–25.3°C and annual precipitation of 1387–2791 mm (Yan and Liang 2024). Fieldwork was conducted in Nada Town, Danzhou City. The town covers an administrative area of approximately 258.86 km^2^ and has a total population of approximately 325,000. The study area encompasses diverse habitat types, including urban built‐up areas, agricultural fields, and secondary forests, providing ample nesting substrates and foraging habitats for barn swallows. Data were collected across Nada Town during the 2024 breeding season (March–June), when active nests encountered within the town were documented, and their respective nest‐site parameters recorded throughout the town's administrative area.

The other study site was located in Zhalong National Nature Reserve, Qiqihar City, Heilongjiang Province (46°48′–47°31′ N, 123°51′–124°37′ E), situated on the Songnen Plain in northeastern China. The site has a temperate continental monsoon climate, with a mean annual temperature of 0.7°C–4.2°C and annual precipitation of 400–550 mm (Yan and Liang 2024). Fieldwork was conducted in Zhalong Town, Tiefeng District, Qiqihar City. The town covers an administrative area of approximately 564.6 km^2^ and has a total population of approximately 29,000. The study area is dominated by freshwater wetlands with slow‐flowing rivers, extensive reed marshes ( *Phragmites australis* ), and scattered aquaculture ponds, creating a mosaic of open water, emergent vegetation, and rural settlements. During the 2024 breeding season (June–August), active nests encountered within the town were documented, and their respective nest‐site parameters were measured and recorded directly in the field.

The barn swallow is a common summer migrant in China, distributed across all provinces. Migration southward begins in autumn, with only small numbers overwintering in southern Yunnan, Hainan Island, Taiwan, and the Xisha Islands (Zheng 2023). The breeding season in Hainan spans from March to June (Liang et al. 2013), and that in Heilongjiang spans from May to August (Li et al. 2019). Barn swallows typically raise two broods per year, with the first generally from March to June and the second from June to August (Liang et al. 2013; Tian et al. 2024).

### 2.2 Data Collection

At both study sites, we located buildings with active barn swallow nests. In Danzhou, swallows typically nest under the eaves of residential buildings, with one to two active nests per household (free‐standing, unattached house). In Zhalong, nests are more commonly found inside storage buildings and livestock enclosures (e.g., cattle, horse, sheep, and chicken pens), with individual structures often containing 10 or more active nests. Once an active nest was confirmed, we monitored it at regular intervals of approximately 2–3 days throughout the breeding period. At each inspection, we directly examined the interior of the nest and photographed its contents to accurately identify the materials present.

### 2.3 Statistical Analysis

Given the high occurrence of human hair in barn swallow nests and its documented role in entanglement mortality (see Section 3), we classified nest material composition into three categories: no anthropogenic material (hereafter “None”), plastic (hereafter “Plastic”), and human hair (hereafter “Human hair”). To test whether the frequency of each nest material category differed within each geographic population, we conducted pairwise Chi‐square (*χ* ^2^) tests for each pair of material categories within each population (northern and southern), with *p*‐values adjusted for multiple comparisons using Bonferroni correction (*p*_adjust_). To test whether the proportion of each nest material category differed between geographic populations, we conducted two‐proportions *z*‐tests, again with Bonferroni‐corrected *p*‐values for multiple comparisons. All statistical analyses were conducted using R software (R Core Team 2023).

## 3 Results

**FIGURE 1:**
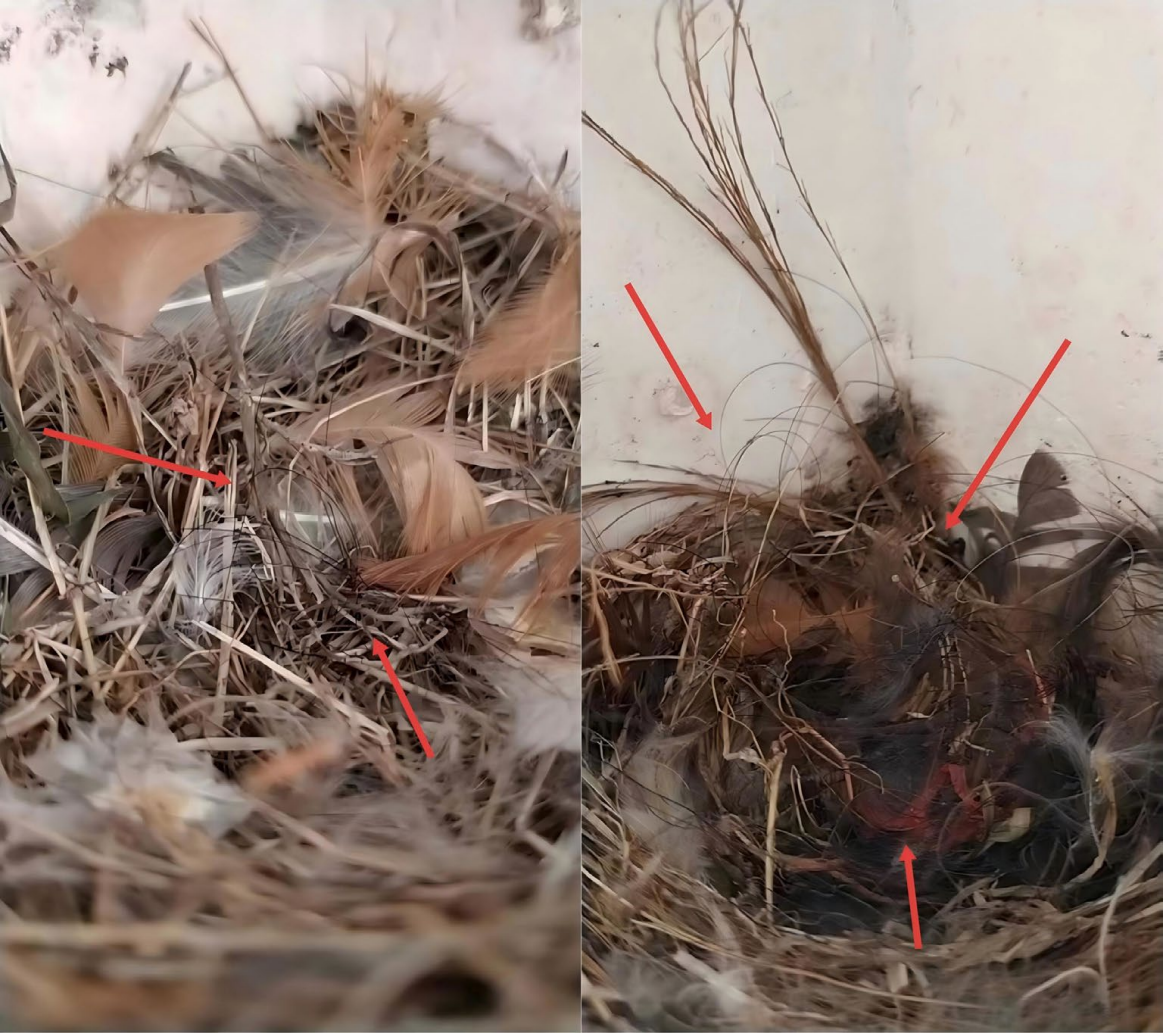
Anthropogenic materials (human hair and plastic) found in barn swallow nests. Red arrows pinpoint human hair and plastic in the nest.

**FIGURE 2:**
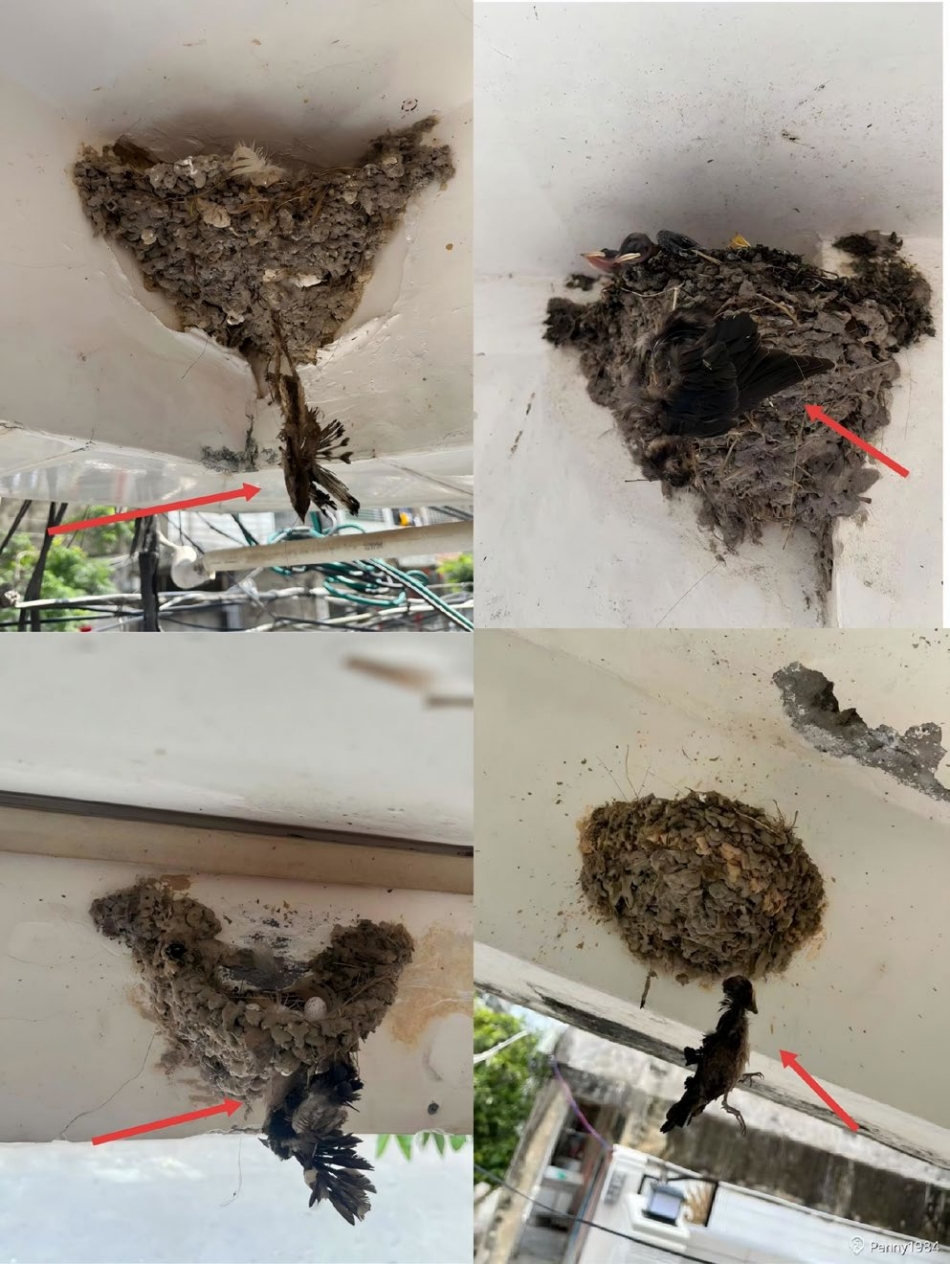
Cases of entanglement mortality caused by human hair in barn swallow nests from the Hainan population. Red arrows pinpoint the deceased barn swallows.

**FIGURE 3:**
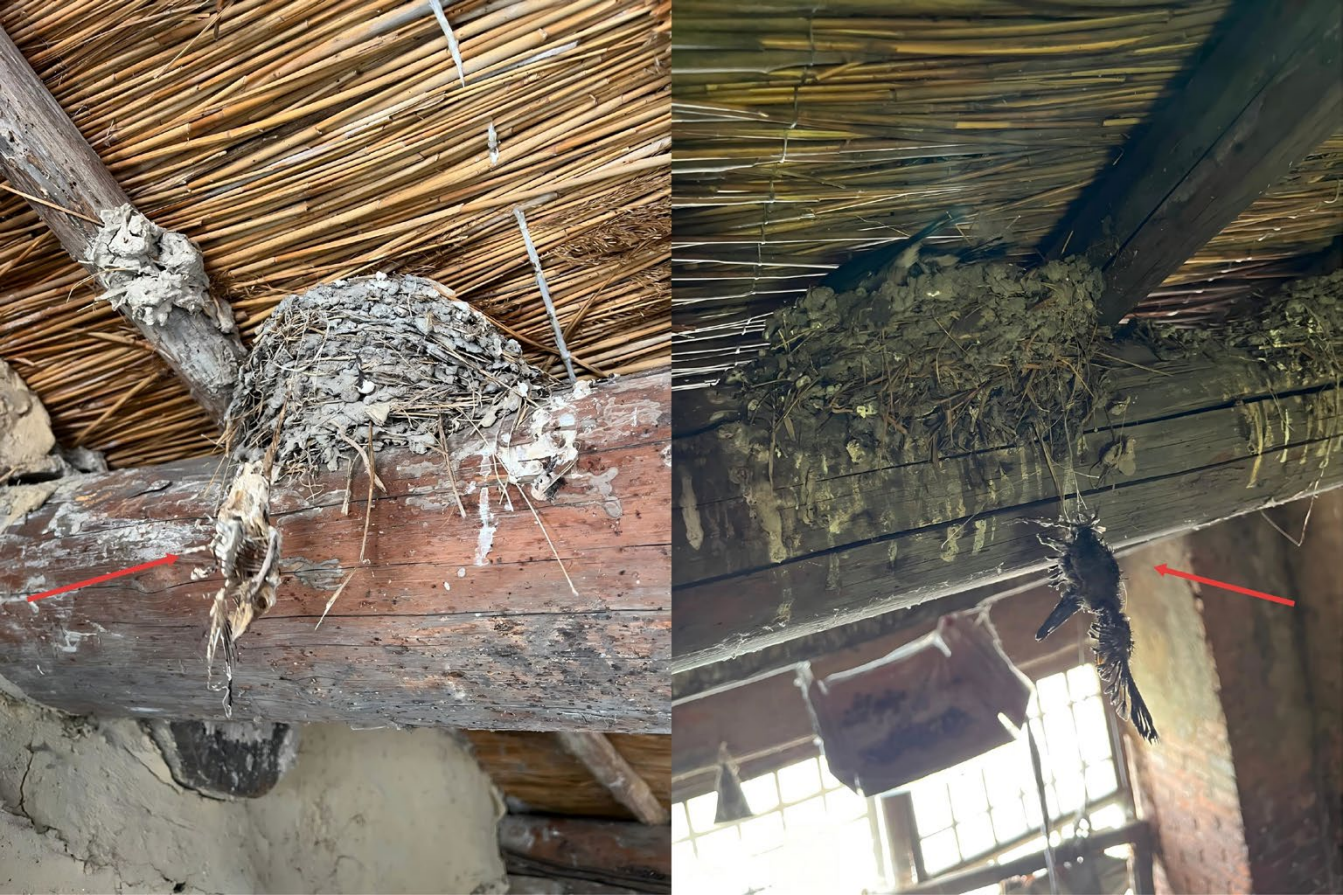
Entanglement mortality caused by fishing line in barn swallow nests from the Heilongjiang population. A total of three cases were documented in the field; two representative cases are shown here. Red arrows pinpoint the deceased barn swallows.

We surveyed a total of 193 b swallow nests (123 in Hainan, 70 in Heilongjiang). Plastic was found in 42 nests (21.8%; 35 in Hainan, 7 in Heilongjiang), and human hair in 43 nests (22.3%; 42 in Hainan, 1 in Heilongjiang). Of these, 11 nests (5.7%, all in Hainan) contained both plastic and human hair (Figure 1). We recorded seven cases (3.6%) of entanglement mortality in breeding adults or nestlings caused by human hair or plastic: four cases (2.1%) in Hainan involving human hair (Figure 2) and three cases (1.5%) in Heilongjiang involving fishing line (Figure 3). All individuals were found deceased and stiff at the time of discovery.

Within‐population pairwise comparisons showed that in the Hainan population, no significant differences were found in the frequency of any pair of nest material categories: Human hair versus None (*χ* ^2^ = 2.3, df = 1, *p*_adjust_ = 0.395), Human hair versus Plastic (*χ* ^2^ = 0.6, df = 1, *p*_adjust_ = 1.000), and None versus Plastic (*χ* ^2^ = 5.3, df = 1, *p*_adjust_ = 0.065). In the Heilongjiang population, Human hair was significantly less frequent than None (*χ* ^2^ = 59.1, df = 1, *p*_adjust_ < 0.001), Plastic was significantly less frequent than None (*χ* ^2^ = 43.8, df = 1, *p*_adjust_ < 0.001), and there was no significant difference in frequency between Hair and Plastic (*χ* ^2^ = 4.5, df = 1, *p*_adjust_ = 0.101) (Table 1).

**TABLE 1:** Chi‐square test results for pairwise comparisons of the frequency of the three nest material categories within each geographic population.

Site	Comparison	*χ* ^2^	*p*	Adjusted *p*
Hainan	Human hair‐None	2.3	0.132	0.395
	Human hair‐Plastic	0.6	0.425	1.000
	None‐Plastic	5.3	**0.022**	0.065
Heilongjiang	Human hair‐None	59.1	< **0.001**	< **0.001**
	Human hair‐Plastic	4.5	**0.034**	0.101
	None‐Plastic	43.8	< **0.001**	< **0.001**

*Note:* Bold values indicate significant differences (*p* < 0.05).

Between‐population comparisons showed significant differences in the proportion of each material category. Both Human hair (*χ* ^2^ = 23.0, df = 1, *p*_adjust_ < 0.001) and Plastic (*χ* ^2^ = 6.4, df = 1, *p*_adjust_ = 0.035) occurred at significantly higher proportions in the Hainan population than in the Heilongjiang population (Table 1).

## 4 Discussion

This study documents the high prevalence of anthropogenic materials in barn swallow nests (38.3%), with human hair, as an unusual anthropogenic material, occurring at a rate (22.3%) comparable to or exceeding that of more commonly reported plastic (21.8%), particularly in the tropical Hainan population. We report the first known cases of entanglement mortality in barn swallows caused by human hair incorporated into nests. The observed difference in nest material composition between the two study populations is directionally consistent with the prediction that anthropogenic debris would be more prevalent in areas of higher human population density. However, given the opportunistic nature of our sampling design, this association remains descriptive. Notably, during nest inspections in Hainan, nestlings were frequently found with human hair tangled around their feet, requiring careful removal before the bird could be extracted. In some cases, nestlings were found with human hair in their bills or had partially ingested it, necessitating manual extraction to prevent harm.

Plastic entanglement and ingestion have been widely documented across marine megafauna globally (Kühn and van Franeker 2020). However, the accumulation of anthropogenic litter in bird nests represents a comparatively recent and understudied form of wildlife–litter interaction, with few studies reporting injury or mortality attributable to nest materials (Votier et al. 2011; Restani 2023). On Grassholm Island in Wales, United Kingdom, northern gannets ( *Morus bassanus* ) incorporate plastic into their nests; synthetic plastic rope caused the entanglement of an estimated 33–109 birds per year, totaling 525 individuals over 8 years, the majority of which were nestlings (Votier et al. 2011). In the Yellowstone River, Montana, United States, between 2014 and 2022, polypropylene baling twine was present in an average of 44.2% of osprey nests annually, resulting in the entanglement of an average of 3.4% of nestlings per year (Restani 2023). In southern Portugal (Alentejo and Algarve), polypropylene baling twine in white stork nests was the principal cause of nestling entanglement mortality (Heinze et al. 2025). The entanglement of barn swallows in hair incorporated into nests has been documented previously, though such reports have exclusively involved animal‐derived hair. Hendricks and Martin (1972) reported horsehair as a major mortality factor for nestling barn swallows in New Mexico, USA, where all 21 nests examined contained horsehair and at least two nestlings died from entanglement. Similarly, Knight and Ryan (1980) described a case in Washington, USA, in which an adult barn swallow was found strangled by a loop of horsehair suspended from the nest rim. These early observations established that hair—particularly horsehair—can pose a significant entanglement risk to this species. Although all previously documented cases of entanglement mortality caused by nest materials involved plastic and animal‐derived hair, our study identifies human hair as a previously unrecognized anthropogenic material capable of causing entanglement mortality in birds. Of the 193 barn swallow nests we examined, 43 (22.3%) contained human hair, with an especially high rate of 34.1% (42 of 123 nests) in Hainan. To our knowledge, this is the first study to report entanglement mortality in barn swallows caused by human hair in nest materials. Human hair is composed largely of keratin, a highly resistant protein that takes years to decompose and re‐enter natural biogeochemical cycles (Gupta 2014). Human hair is recognized as a globally important potential biowaste, and its improper disposal is associated with a range of environmental problems (Mondal et al. 2020). Our findings demonstrate that environmental human hair can directly cause bird mortality, contributing to a more complete understanding of the potential impacts of discarded human hair on wildlife.

The likelihood of birds incorporating anthropogenic materials into nests is positively associated with the degree of human influence on the surrounding environment (Jagiello et al. 2019, 2023). For instance, during white stork breeding in Spain, the probability of using anthropogenic nest materials increased with impervious surface area, and the quantity of such materials increased with human footprint index (HFI) (Jagiello et al. 2023). The influence of HFI on the likelihood of anthropogenic materials appearing in nests has also been demonstrated in three swift species (common swift [ *Apus apus* ], pallid swift [ *A. pallidus* ], and alpine swift [ *Tachymarptis melba* ]) (Luna et al. 2024). In light‐vented bulbul ( *Pycnonotus sinensis* ) nest sites, both the proportion and the total mass of anthropogenic nest materials increased with increasing urbanization (Chen et al. 2024). Our results indicate that the two barn swallow populations are subject to different levels of environmental pollution pressure, as reflected in significant differences in the proportion of anthropogenic nest materials. However, the nest sites surveyed within each population were geographically clustered (within a 30 km radius in Heilongjiang and 5 km radius in Hainan), and we lack data at a broader spatial scale to quantify the relationship between human disturbance and nest material composition, which needs further investigations. Several studies have suggested that birds may serve as indicator species for plastic contamination in the environment (Jagiello et al. 2019). Seabirds, for example, have been proposed as biological indicators of pollution in estuarine and marine environments (Burger and Gochfeld 2004), and the nest‐collecting behavior of yellow‐legged gulls ( *Larus michahellis* ) has been used to indicate inadequate urban waste management (Galimany et al. 2023). Beyond aquatic and terrestrial environments, birds are uniquely positioned to serve as indicators of atmospheric plastic pollution: swifts, given their highly aerial lifestyle, have been proposed as sentinels of atmospheric pollution concentrations through both entanglement debris on their bodies and anthropogenic materials in their nests (Luna et al. 2024). Given the wide distribution of barn swallows and their aerial foraging behavior, using fecal analysis to monitor airborne anthropogenic material pollution may represent a promising approach.

In summary, our study documents the high prevalence of anthropogenic materials (plastic and human hair) in barn swallow nests and significant variation between geographic populations. Additionally, we report for the first time that hair incorporated into nests can cause entanglement mortality in barn swallows, indicating that ubiquitous human hair waste is a potential threat to birds. As urbanization continues to expand, the incorporation of anthropogenic materials into bird nests is likely to become increasingly prevalent, highlighting the need for a comprehensive and in‐depth understanding of the potential future consequences of this ecological trap for avian populations.

## Author Contributions

**Cong Peng:** investigation (lead), methodology (lead), visualization (equal), writing – original draft (equal). **Kui Yan:** investigation (equal), resources (equal). **Sidan Lin:** data curation (equal), formal analysis (equal), visualization (equal), writing – review and editing (equal). **Wei Liang:** conceptualization (lead), funding acquisition (lead), supervision (lead), validation (equal), writing – review and editing (equal).

## Funding

This work was supported by the National Key R & D Program of China (2023YFF1304600).

## Ethics Statement

The experiments comply with the current laws of China. No special permit was required for this study as it was not involved in animal or plant collection.

## Conflicts of Interest

The authors declare no conflicts of interest.

## Supporting information


**Table S1:** ece373592‐sup‐0001‐TableS1.xlsx.

## Data Availability

Data used for this study are provided as Supporting Information and can be found at https://figshare.com/s/5f8f9f8167818a2822c3 (doi: https://doi.org/10.6084/m9.figshare.31563076).
